# Antidepressant-like effects of methanolic extract of *Bacopa monniera* in mice

**DOI:** 10.1186/s12906-015-0866-2

**Published:** 2015-09-25

**Authors:** Abdul Mannan, Ariful Basher Abir, Rashidur Rahman

**Affiliations:** Department of Pharmacy, Stamford University Bangladesh, 51, Siddeswari Road, Dhaka, 1217 Bangladesh; Department of Pharmacy, Faculty of Biological Science and Technology, Jessore University of Science and Technology, Jessore, 7408 Bangladesh

**Keywords:** *Bacopa monniera*, Imipramine hydrochloride, Antidepressant-like effect, Forced swimming, Tail suspension

## Abstract

**Background:**

*Bacopa monniera* has been used as a cure for various ailments that include anxiety, epileptic disorders, dementia, blood purifier, cough and rheumatism, and some important local uses of the plant are in dermatitis, anemia, diabetes, promote fertility and prevent miscarriage for many years in Bangladesh. According to this background, the aim of the study was to evaluate the antidepressant-like effect of the methanolic extract of *B. monniera* (MEBM) in different behavioral models such as forced swimming test (FST), measurement of locomotor activity test (MLAT) and tail suspension test (TST) on mice after two weeks treatment.

**Methods:**

Mice were divided into five groups (*n* = 5/group): control group (deionized water), standard group where Imipramine hydrochloride (30 mg/kg) was used as standard drug and three test groups where three doses of the methanolic extract of *B. monniera* (MEBM) (50, 100, and 200 mg/kg) was used for two weeks treatment. All the drug and test samples were administered via gavage through oral route. To assess the antidepressant-like effect of MEBM forced swimming test (FST), tail suspension test (TST) and measurement of locomotor activity test (MLAT) have been done in mice.

**Results:**

The results showed that a strong and dose-dependent antidepressant effects in different mice models. The main findings of the MEBM significantly reduced the duration of immobility times in the forced swimming test (*p* < 0.001). Likewise, the extract significantly decreased the immobility time in the tail suspension test (*p* < 0.001). Moreover, we employed an additional measurement of locomotor activity test to check the motor stimulating activity of the MEBM. The extract also significantly increased the locomotion, rearing and defecation effects in comparison to the control group (*p* < 0.001).

**Conclusion:**

The present results clearly demonstrate that the methanolic extract of *B. monniera* possesses antidepressant-like activity in the animal behavioral models. The current study warrants further investigation into identification of the active compounds in herbal medicines, in particular extract of *B. monniera* with antidepressant-like effects.

## Background

Depression is the second leading psychiatric disorder where 21 % of the world population suffers from this disease [1]. The age range is markedly decreasing from 40–50 years age range to 25–35 years age range which observed worldwide [2]. In last few decades, several drugs have been discovered to treat depression such as tricyclic antidepressants, monoamine oxidase inhibitors [3] and selective serotonin reuptake inhibitors (SSRI). But unfortunately, all of the drugs have serious side effects including insomnia, anxiety, weight gain etc. It is well known that nature is the best and safe source for all medicine. So it becomes worth to search for a new antidepressant drug from natural source with less side effects (It is assume that a drug from natural source could have less side effects) and complications [4].

*Bacopa monniera* (Family: Scrophulariaceae), commonly known as Brahmi, is an aquatic herbs distributed throughout the warmer regions of the world including Bangladesh. It grows in wet, damp, marshy areas and possesses a wide range of medicinal values including memory enhancement, improvement of cognitive disorders, focusing and alertness as well as reduces anxiety [5, 6]. In Bangladesh, this plant is extensively used in the traditional medicine system as potent therapeutic agent as a neurological tonic to enhance intellectual development [7], to treat epilepsy [8], cardiac [9], respiratory [10] and digestive [11] disorders, toothache and purifies blood. In some parts of this country Brahmi is used to treat rheumatism and to prevent miscarriage [12]. Conversely, *B. monniera* is associated with gastrointestinal side-effects, specifically increased bowel movements, nausea, and abdominal cramping [13]. Toxicology studies of the extract used in the current study—BacoMind,™—have not shown gastrointestinal reactions in rats [14] and safety and tolerability studies of BacoMind™ in human volunteers reported only mild gastrointestinal reactions in 3 of 23 participants that subsided instinctively. Possibly the higher incidence of gastrointestinal reactions in the current study was due to the older age of participants, lowering their capacity to tolerate Bacopa [13]. There are several reports revealing that Brahmi contains flavonoids [15], alkaloids, glycosides, saponins and sterols [16]. Some researchers isolated some of these phytochemicals such as Brahmin, nicotine, herpestine, des-saponin glycosides-triterpenoid saponins like Bacosides A & B. It has been found that the phytochemicals like 3-(a-L-arabinopyranosyl)-O-b-D-glucopyranoside-10 and 20-dihydroxy-16-keto-dammar-24-ene (Bacosides A & B) are the major compounds that may exhibit neuropharmacological activities by directly acting on the neurotransmeter’s level [17, 18]. The other chemical constituents reported for this plant includes A1 & A3 [19, 20], hersaponin [21], betulic acid, monnierin [22], herpestin and brahmine [23], luteolin-7-glucoside, glucoronyl-7-apigenin and gluucoronyl-7-luteonin, common phytosteroids [7]. Due to the diverse pharmacological actions of Brahmi, research tried to investigate and validate its ethnomedical uses based on the advanced research techniques. They found Brahmi as a potent agent with antioxidant [24], antiulcerogenic [25], cognitive enhancer [26], anti-inflammatory, anxiolytic [27], analgesic, antipyretic, and sedative [26] properties. However, the neuropharmacological activity of this plant is not investigated extensively which influenced us to design our study. The present study investigated the antidepressant-like effect of MEBM in different behavioral model of depression in mice.

## Methods

### Plant material and extraction

The leaves of *B. monniera* were collected from the village Khanpur, Bogra, Bangladesh in June, 2014. The collected samples were then identified by Sarder Nasir Uddin, Senior Scientific Officer, Bangladesh National Herbarium, Dhaka, Bangladesh. A voucher specimen (DACB: 38106) has been deposited in the Herbarium for further reference. About 250 g of powdered material have to be soaked in 800 mL methanol at 25 ± 2 °C for 72 h in a beaker and mixture needs to be stirred every 18 h using a sterile glass rod. Filtrate was obtained 3 times with the help of Whatman No. 1 filter paper and sterilized cotton filter. The solvent was removed by rotary evaporator and 10.56 g extract (Yield 4.22 %) was obtained. This crude extract was used for the investigations of antidepressant-like effect of the methanolic extract of *B. monniera* in mice.

### Animals

Swiss Albino mice (20–25 g) were collected from the Animal Research Branch of the International Center for Diarrheal Disease and Research, Bangladesh (ICDDR, B). Animals were maintained under standard environmental conditions (temperature: 25.0 ± 2.0 °C, relative humidity: 55–65 % and 12 h light/ dark cycle). Husk and excreta were removed from the cages every day. Pellets of mice foods, provided by ICDDR, B were given to the mice with fresh water *ad libitum* during acclimatization period. The animals were acclimatized to the laboratory environment for a period of 14 days prior to performing the experiments into five groups such control, standard and three tests groups (50, 100 and 200 mg/kg). The animals were fasted overnight before the experiments. All the experimental animals were treated following the Ethical Principles and Guidelines for Scientific Experiments on Animals (1995) formulated by The Swiss Academy of Medical Sciences and the Swiss Academy of Sciences. All experimental rules were approved by the Institutional Animal Ethical Committee (SUB/IAEC/14.01) of Stamford University Bangladesh.

### Drugs and treatments

The described experiments used chemicals and drugs such as methanol (Merck, Germany), and Imipramine hydrochloride (Sandoz, Novartis Bangladesh Ltd). The standard drug Imipramine hydrochloride (30 mg/kg) was used in antidepressant activity tests. The sample of methanolic extract of *B. monniera* and standard drug Imipramine hydrochloride were prepared by dissolving in deionized water at the doses of 50, 100, and 200 mg/ kg for sample extract and 30 mg/kg for standard drug respectively. The test and standard groups were received MEBM and drugs orally 30 min before the experiments, whereas the control group received 0.1 mL/mouse deionized water. All the groups received drugs and samples via gavage. Before the final experiments, all the groups of animals treated for two weeks. All the chemicals and the drugs were analytical graded and highly purified.

### Antidepressant activity tests

#### Forced swimming test (FST)

Forced swimming test or behavioral despair test for measuring the susceptibility to negative mood of mice’s threat of drowning, commonly used to measure the effectiveness of antidepressant agents [28]. For assessing the antidepressant activity of the FST is widely used for pharmacological model. This method was adapted on the observation of animals exposed to a situation of forced swimming, in which they become passive and immobile after a period of vigorous activity (struggling), producing only the movements required to keep their heads above the water. The FST was performed according to the method of Porsolt [29, 30] with some modifications. Mice were divided randomly into control, extract and Imipramine hydrochloride. Each group was contained 5 mice. Test solutions were administered once daily between 1 and 3 p.m. over a period of 14 d. Mice were placed in an acrylic cylinder (45 cm height = 20 cm diameter) filled with water at 25 ± 1 °C to a depth of 17 cm for 15 min (pre-test session) after 14 d treatment. Twenty-four hours after the pre-test session, the animals were once again exposed to the same conditions for 5 min (test session). Between the pre-test session and main session drug solutions were administered orally three times as follows: just after the pre-test session, 5 h before the main test, and 1 h before the main test. A mouse was judged immobile if it remained floating in the water, except for small movements to keep its head above the water. The FST was performed between 1 and 3 p.m. for 5 min by observers [31].

### Measurement of locomotor activity test (MLAT)

The locomotor activity test needs to be performed according to the method of Carlini [32] with minor modifications. This test is designed to measure the mobility of mice. A group of 25 mice was divided at random into five groups and orally administered with control, extract or Imipramine hydrochloride 30 min before the experiment. Mice were placed in an open field apparatus composed of an arena 40 cm in diameter divided into 16 approximately equal areas. For open field observations, each mouse was individually placed in the center of the arena 15 h after the last treatment. Hand-operated counters were employed to score the following behavioral parameters: locomotion (number of line crossings), rearing frequencies (number of times seen standing on hind legs), and number of defecations within 5 min. Open field observations was made between 8 and 10 a.m [31].

### Tail suspension test (TST)

For screening antidepressant effect and other class of psychotropics a simple, rapid and reliable method is TST. The test is designed to assay mood level by measuring the immobility time which indicate change in mood. This method was employed on the observation that a mouse suspended by the tail shows alternate agitation and immobility which is indicative of a state of depression. TST induced immobility is reduced by a large no of clinically active and atypical antidepressant effect [33]. The TST was performed according to the method [34] with slight modifications. 25 mice were treated with control, extract or Imipramine hydrochloride and were placed in the middle of the stand. Two stands, each with a clamp located 22 cm from the floor, were placed at intervals of 23 cm. A mouse was hung 5 cm from the end of its tail on a stand, and observed for 6 min. The TST was performed between 1 and 3 p.m. Immobility time was evaluated by observers [31].

### Statistical analysis

The results are presented as mean ± SEM. The statistical analysis was performed using one way analysis of variance (ANOVA) followed by Dunnett’s post hoc test as appropriate using SPSS 11.5 software. Differences between groups were considered significant at a level of *p* < 0.001.

## Results

**Fig. 1:**
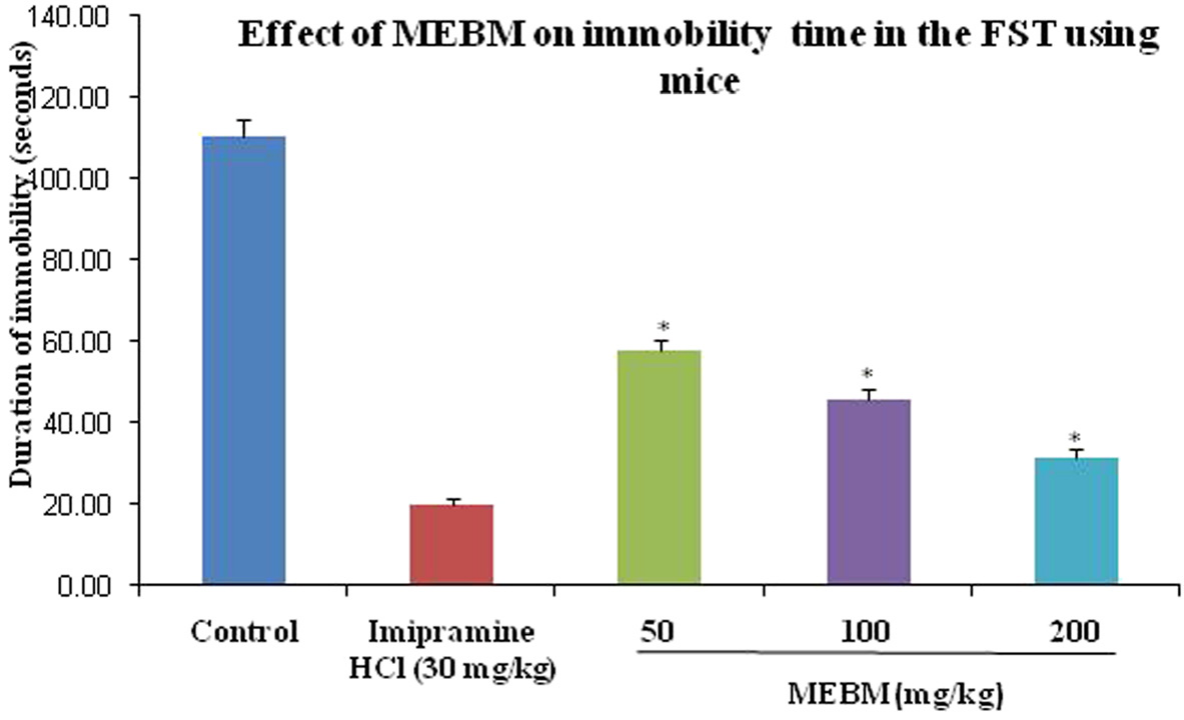
Graphical representation of antidepressant effect of *B. monniera* extract on forced swimming test of immobility time in mice

**Fig. 2:**
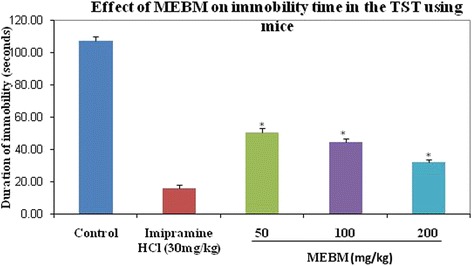
Graphical representation of antidepressant effect of *B. monniera* extract on locomotor activity test of locomotion in mice

The methanolic extract of B. *monniera* showed antidepressant-like effects in prophetic animal models, namely forced swimming, measurement of locomotor activity and tail suspension tests. MEBM (50, 100 and 200 mg/kg body weight) or the synthetic antidepressant drug, Imipramine hydrochloride (30 mg/kg), was orally administered to the mice once daily for 14d. There was no difference in body weight gains after 14d among test treatment groups (Table 1). The extract (50, 100 and 200 mg/kg body weight) significantly reduced the duration of immobility time in the forced swimming test after 14d daily treatment (Table 2 and Fig. 1). Dunnett’s post hoc analysis demonstrated that the test treatments significantly decreased the immobility time in comparison to the control group (*p* < 0.001). Likewise, the extract reduced the duration of immobility time in the tail suspension test (Table 3 and Fig. 2). Post hoc analysis confirmed that the extract significantly decreased the immobility time in comparison to the control group (*p* < 0.001).

**Table 1:** Effects of methanolic extract of *B. monniera* (MEBM) in forced swimming test in body weight (g) gain of mice

Treatment	Doses (mg/kg)	Day 1	Day 14
Deionized water	0.1 mL/mice	22.20 ± 0.86	28.78 ± 1.07
Imipramine hydrochloride	30	23.60 ± 1.12	27.77 ± 1.51
MEBM	50	24.05 ± 1.01	26.11 ± 0.98
MEBM	100	24.54 ± 1.40	24.66 ± 2.99
MEBM	200	24.08 ± 1.07	25.85 ± 1.65

Values are presented as mean ± SEM (*n* = 5). MEBM = Methanolic extract of *B. monniera*

**Table 2:** Antidepressant effects of methanolic extract of *B. monniera* in forced swimming test

Treatment	Doses (mg/kg)	Immobility Time (s)
Deionized water	0.1 mL/mice	110.60 ± 3.88
Imipramine hydrochloride	30	20.00 ± 1.58*
MEBM	50	57.60 ± 2.65*
MEBM	100	45.60 ± 2.73*
MEBM	200	31.40 ± 2.42*

Values are presented as mean ± SEM (*n* = 5). MEBM = Methanolic extract of *B. monniera*

*p < 0.001 compared with the control group (Dunnett’s test)

**Table 3:** Antidepressant effects of methanolic extract of *B. monniera* in tail suspension test

Treatment	Doses (mg/kg)	Immobility Time(s)
Deionized water	0.1 mL/mice	107.40 ± 2.65
Imipramine hydrochloride	30	16.00 ± 2.28*
MEBM	50	50.80 ± 2.35*
MEBM	100	44.40 ± 2.46*
MEBM	200	32.00 ± 1.87*

Values are presented as mean ± SEM (*n* = 5). MEBM = Methanolic extract of *B. monniera*

*p < 0.001 compared with the control group (Dunnett’s test)

**Fig. 3:**
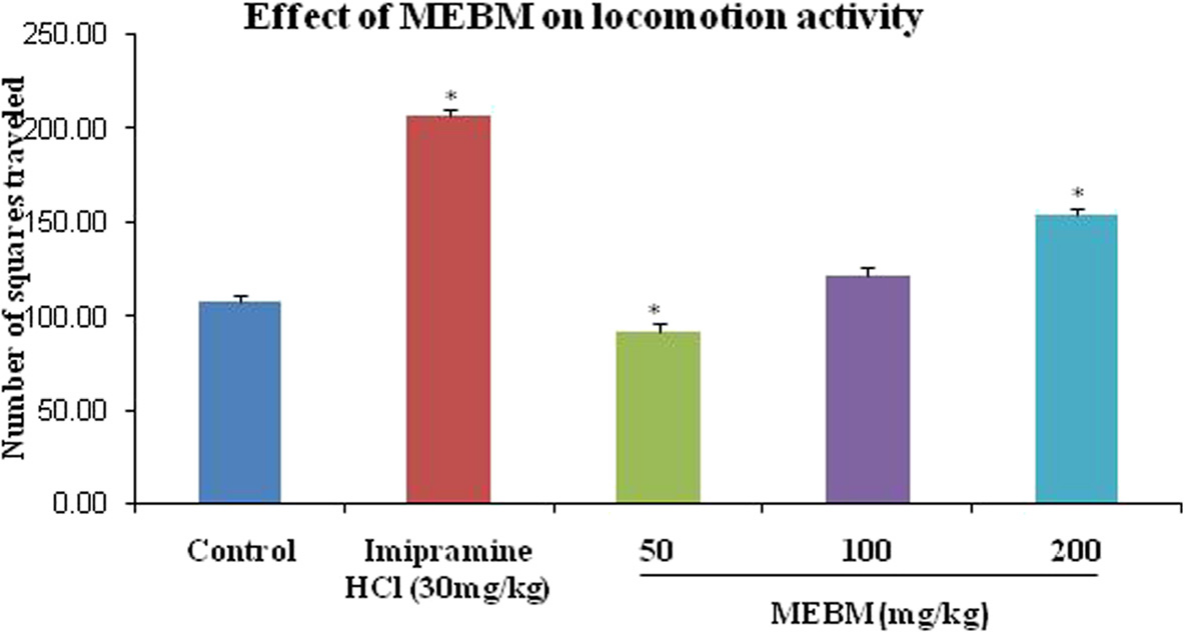
Graphical representation of antidepressant effect of *B. monniera* extract on locomotor activity test of rearing in mice

**Fig. 4:**
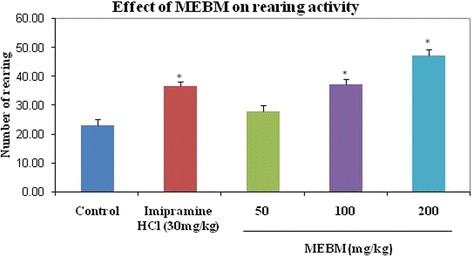
Graphical representation of antidepressant effect of *B. monniera* extract on locomotor activity test of defecation in mice

**Fig. 5:**
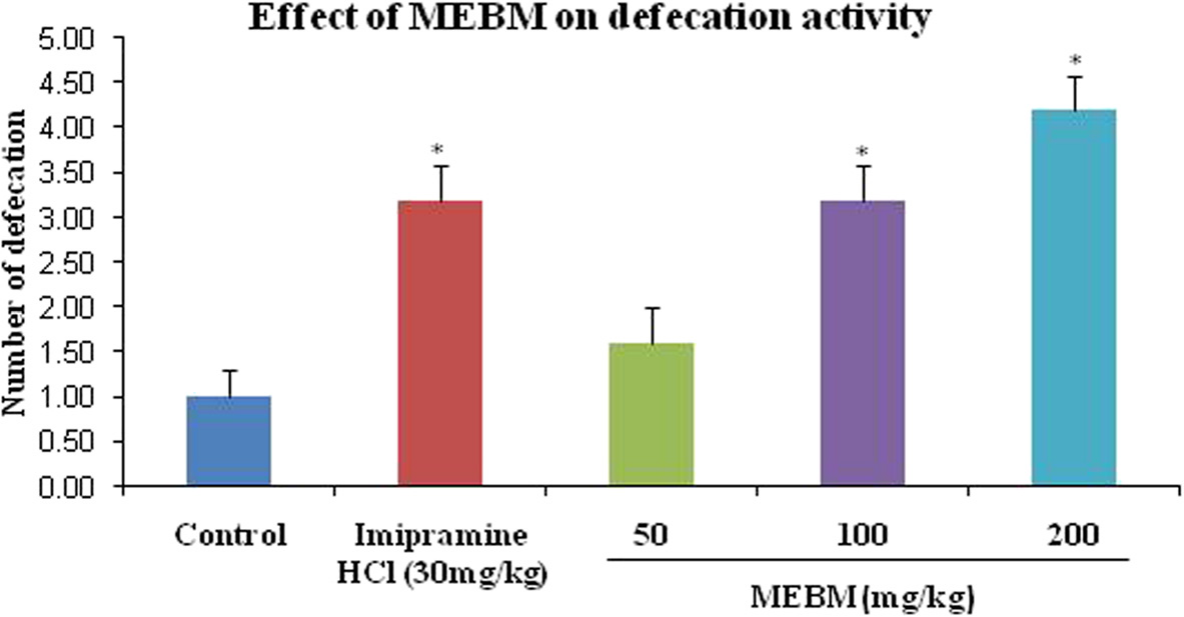
Graphical representation of antidepressant effect of *B. monniera* extract on tail suspension test in mice

The effectiveness of the decreasing immobility in the FST was also shown previously with psycho stimulants, which exert an indiscriminate motor stimulating activity. For this false positive result, we employed an additional measurement of locomotor activity test to check the motor stimulating activity of the MEBM. Administration of the extract of *B. monniera* at doses of 50, 100 and 200 mg/kg for 14 days, active doses in the FST, resulted in no behavioral changes or motor dysfunction in the measurement of locomotor activity test (Table 4 and Figs. 3, 4 and 5).

**Table 4:** Antidepressant effects of methanolic extract of *B. monniera* in measurement of locomotor activity test

Treatment	Doses (mg/kg)	Locomotion	Rearing	Defecation
Deionized water	0.1 mL/mice	107.80 ± 2.92	23.00 ± 2.02	1.00 ± 0.31
Imipramine hydrochloride	30	206.60 ± 3.01*	36.60 ± 1.60*	3.20 ± 0.37*
MEBM	50	91.40 ± 4.25*	27.80 ± 2.13	1.60 ± 0.40
MEBM	100	121.40 ± 4.46	37.20 ± 1.93*	3.20 ± 0.37*
MEBM	200	153.80 ± 3.61*	47.20 ± 2.03*	4.20 ± 0.37*

Values are presented as mean ± SEM (*n* = 5). MEBM = Methanolic extract of *B.monniera*

*p < 0.001 compared with the control group (Dunnett’s test)

As shown in Fig. 3, MEBM (50 and 200 mg/kg) was shown the satisfactory locomotion effect. At the doses of 100 and 200 mg/kg was significantly augmented the good rearing effect of this test (Fig. 4). Extracts of *B. monniera* (100 and 200 mg/kg) was shown good effect of defecation phase (Fig. 5). Moreover, Post hoc analysis also verified that the extract significantly increased the locomotion, rearing and defecation effects in comparison to the control group (*p* < 0.001).

## Discussion

The purpose of this study was assessed the antidepressant-like effect of MEBM using animal behavioral models. A major problem in the screening for new antidepressant effect is the establishment of a valid animal model able to sufficiently and accurately identified diverse depressant treatments, without making errors of omission [35]. In that case, the forced swimming and tail suspension tests are widely accepted behavioral models for the assessment of antidepressant activity. The characteristic behavior evaluated in these tests, termed immobility, has been considered to reflect behavioral despair similar to that seen in human depression, and it is well known that antidepressant drugs are able to reduce the immobility time in rodents [30]. It is interesting to note that the immobility shown by mice when subjected to unavoidable stress such as forced swimming test is thought to reflect a state of despair or lowered mood, which is thought to reflect depressive disorders in humans. In addition, the immobility time is reduced by treatment with antidepressant drugs [36]. There is a significant correlation between the clinical efficacy of antidepressant drugs and their potency in the FST, this was not found in any other model [34, 36]. Interestingly, our data indicate that higher doses of plant extracts were more effective than smaller doses both in forced swimming and tail suspension tests.

Based on our present study, antidepressant-like effect of MEBM in all the classic models of depressants, where it was found to possess antidepressant-like activity comparable to the standard drug Imipramine hydrochloride. Imipramine hydrochloride acts by inhibiting norepinephrine (NE) reuptake and has been used as a standard drug in majority studies. The beneficial effect of Imipramine hydrochloride in the forced swimming test model seems to be due to increased availability of these neurotransmitters (NE) and serotonin (5HT) at the post synaptic site following reuptake inhibition [37].

Initial hypothesis of depression has been formulated about 40 years ago, proposing that the main symptoms of depression due to functional deficiency of cerebral monoaminergic transmitters such as (NE), 5HT, and dopamine (DA) located at synapses [38]. Some studies have also shown the adaptogenic effect of the plant extract via normalization of the various stress parameters and monoaminergic levels which may provide a clue that the extract is bringing their possible antidepressant-like effect through restoration of normal monoaminergic neurotransmitters [39].

Brahmi contains a natural phytonutrients which is known as bacosides. This is responsible for improving vital neurotransmitters activities which happen in memorization and information process and may be helpful in depression [40]. The action of the triterpinoid saponins and the bacosides A and B has resulted in the enhancement of the nerve impulse transmission. Neurochemical assays suggested that treatment by bacosides or bacopasides I improved brain antioxidant activity to varying degrees after the behavioral despair test. These findings indicated that the antidepressant-like effect of bacopasides I might be related to both antioxidant activation and noradrenergic activation [41]. Three new triterpene glycosides, bacopasides VI-VIII, together with three known analogues, bacopaside I (1) , bacopaside II (2) and bacopa saponsin C (3), were isolated from the whole plant of *B. monniera*. Compounds 1, 2 and 3 were shown antidepressant activity when tested on forced swimming and tail suspension in mice, respectively [42].

Recently, oxidative stress was linked with the pathophysiology of major depression, with significant correlations being found between the severity of depression and erythrocyte superoxide dismutase/lipoperoxidation levels [43]. Meanwhile, treatment with antidepressants reduces the oxidative stress related to depressive disorder [44, 45]. Additionally, some species such as *Bacopa monneira*, *Withania somnifera* and *Asparagus racemosus*, all of which are reported to have antidepressant-like properties, also possess antioxidant activity [46–48]. Therefore , it is possible that the antioxidant activity of the methanol extract from *B. monniera* may contribute to its antidepressant-like effect. However, different kinds of the research study must needed to elucidate the mechanism of action of *B. monniera* in the CNS, the pattern of effects were observed in these experiments suggest the involvement of the norepinephrine neurotransmitters system on its antidepressant-like effects.

## Conclusions

In the present investigation, we have reported antidepressant-like effect of MEBM in all the classic models such as forced swimming test (FST), measurement of locomotor activity test (MLAT) and tail suspension test (TST), where it was found to possess significant antidepressant-like activity comparable to the standard drug Imipramine hydrochloride. Different kinds of the research study must needed to elucidate the mechanism of action of *B. monniera* in the CNS, the pattern of effects were observed in these experiments suggest the involvement of norepinephrine neurotransmitters system on its antidepressant-like effect. The present study also warrants further investigation into identification of the active compounds in herbal medicines, in particular extract of *B. monniera* with antidepressant-like effects.

## Abbreviation

MEBM: Methanolic extract of *Bacopa monniera*
FST: Forced swimming test
ICDDR, B: International Center for Diarrheal Disease and Research, Bangladesh
TST: Tail suspension test

## References

[1] Schechter LE, Ring RH, Beyer CE, Hughes ZA, Khawaja X, Malberg JE, Rosenzweig-Lipson S (2005). Innovative approaches for the development of antidepressant drugs: current and future strategies. NeuroRx.

[2] Nemeroff CB, Owens MJ (2002). Treatment of mood disorders. Nat Neurosci.

[3] Belmaker RH, Agam G (2008). Major depressive disorder. N Engl J Med.

[4] Zhang ZJ (2004). Therapeutic effects of herbal extracts and constituents in animal models of psychiatric disorders. Life Sci.

[5] Satyavati GV, Raina MK, Sharma M (1976). Chemical investigation of *Herpestis monniera* Linn (Brahmi). Indian J Pharmacology.

[6] Barrett SCH, Strother JL (1978). Taxonomy and natural history of *Bacopa* in California. Syst Bot.

[7] Singh HK, Dhawan BN (1982). The effect of *Bacopa monniera* Linn. (*Brahmi)* extract on avoidance responses in rat. J Ethanopharmaco.

[8] Sen S, Chakraborty R, Sridhar C, Reddy YSR, De B (2010). Free radicals, antioxidants, Diseases and phytomedicines: Current status and Future prospect. Int J Pharma Sci Rev and Res.

[9] Valko M, Leibfritz D, Monco J, Cronin MTD, Mazur M, Telser J (2007). Free radicals and antioxidants in normal physiological functions and human disease. Int J Biochem and Cell Bio.

[10] Mohan H (2010). Cell Injury and cellular Adaptations. Textbook of Pathology.

[11] Kumar V, Abbas AK, Fausto N, Robbins and Cotran (2009). Cellular Adaptations, cell injury and cell death. Pathologic basis of disease.

[12] Sudharani D, Krishna KL, Deval K, Safia AK, Priya (2011). Pharmacological profile of *Bacopa monnieri*: A review. Int J Pharma.

[13] Morgan A, Stevens J (2010). Does Bacopa monniera improve memory performance in older persons? Results of a randomized, placebo-controlled, double-blind trial. J Alter Comple Med.

[14] Allan J, Damodaran A, Deshmukh NS, Goudar KS, Amit A (2007). Safety evaluation of a standardized phytochemical composition extracted from Bacopa monnieri in Sprague–Dawley rats. Food ChemToxicol.

[15] Chaterjee N, Rastigi RP, Dhar ML (1963). Chemical examination of *Bacopa monniera* Wettst. Part I: Isolation of chemical constituents. Indian J Chem.

[16] Bose KC, Bose NK (1931). Observations on the actions and uses of *Herpestis monniera*. J Ind Med Assoc.

[17] Chaterjee N, Rastogi RP, Dhar ML (1965). Chemical examination of *Bacopa monneri*. Indian J Chem.

[18] Basu N, Rastogi RP, Dhar ML (1967). Chemical examination of *Bacopa monniera* westst: Part III-Bacoside B. Indian J Chem.

[19] Jain P, Kulshresta DK (1993). Bacoside A1, a minor saponin from *Bacopa monniera*. Phytochemistry.

[20] Rastogi S, Pal R, Kulshreshtha K (1994). Bacoside A3—a triterpenoid saponin from *Bacopa monniera*. Phytochemistry.

[21] Shashtri MS, Dhalla NS, Malhotra CL (1959). Chemical investigation of *Herpestis monniera* Linn (Brahmi). Indian J Pharm.

[22] Basu UP, Dutta T (1967). The structure of monniera. Tetrahedron Lett.

[23] Schulte KE, Rucker G, Etkersch M (1972). Nicotin and 3-formyl-4-hydroxy-2H-pyranaus *Herpestis monniera*. Phytochemistry.

[24] Biswas SK, Das J, Chowdhury A, Karmakar UK, Hossain H (2012). Evaluation of antinociceptive and antioxidant activities of whole plant extract of Bacopa monniera. Res J Med Plant.

[25] Sairam K, RaoCh V, Dora Babu M, Goel RK (2001). Prophylactic and curative effects of *Bacopa monniera* in gastric ulcer models. Phytomedicine.

[26] Nathan PJ, Clarke J, Lioyd J, Hutchison CW, Downey L, Stough C (2001). The acute effect of an extract of *Bacopa monniera* (Brahmi) on cognitive functions in healthy normal subjects. Human Psycho Pharmacol Clin Exp.

[27] Anju Bacopa monnieri – a preliminary study evaluating its anti-stress activity in Swiss albino mice. RJPBCS. 2011, 2: 786–94.

[28] Borsini F, Volterra G, Meli A (1986). Does the behavioral “despair” test measure “despair”?. Physiol Behav.

[29] Porsolt RD, Le Pichon M, Jalfre M (1977). Depression: a new animal model sensitive to antidepressant treatments. Nature.

[30] Porsolt RD, Bertin A, Jalfre M (1977). Behavioural despair in mice: a primary screening test for antidepressants. Acrh Inter Pharmacodyn Ther.

[31] Sakakibara H, Ishida K, Grundmann O, Nakajima JI, Seo S, Butterweck V, Minami Y, Saito S, Kawai Y, Nakaya Y, Terao J (2006). Antidepressant effect of extracts from *Ginkgo biloba* leavesin behavioral models. Biol Pharm Bull.

[32] Carlini EA, de Contar JDP, Silva-Filho AR, Da Silveira-Filho NG, Frochtengarten ML, Bueno OFA (1986). Pharmacology of lemongrass (*Cymbopogon citratus* Stapf). I. effects of teasprepared from the leaves on laboratory animals. J Ethnopharmacol.

[33] Wesołowska A, Nikiforuk A, Stachowicz K, Tatarczyńska E (2006). Effect of the selective 5-HT7 receptor antagonist SB 269970 in animal models of anxiety and depression. Neuropharmacology.

[34] Steru L, Chermat R, Thierry B, Simon P (1985). The tail suspension test: a new method for screening antidepressants in mice. Psychopharmacol.

[35] Willner P (1984). The validity of animal models of depression. Psychopharmacology (Berl).

[36] Porsolt RD. Behavioral despair, Antidepressants: neurochemical, behavioral and clinical perspectives. In: Enna SJ, Malick JB, Richelson E editors. New York: Raven Press. 1981, 121–139.

[37] Pal SN, Dandiya PC (1993). Comparative study of imipramine, maprotiline, fluvoxamine, trazodone and Alprozolam in some animal models of depression. Indian J Pharmacol.

[38] Schildkraut JJ (1965). The catecholamine hypothesis of affective disorders. A review of supporting evidence. Am J Psychiat.

[39] Rai D, Bhatia G, Palit G, Pal R, Singh S, Singh HK (2003). Adaptogenic effect of *Bacopa monniera* (brami). Pharmacol Bio Chem Behave.

[40] Chatterjee M, Verma P, Palit G (2010). Comparative evaluation of *Bacopa monniera* and Panax quniquefolium in experimental and depressive models in mice. Indian J Exp Biol.

[41] Liu X, Liu F, Yue R, Li Y, Zhang J, Wang S, Zhang S, Wang R, Shan L, Zhang W (2013). The antidepressant-like effect of bacopaside I: possible involvement of the oxidative stress system and the noradrenergic system. Pharmacol Biochem Behav.

[42] Zhou Y, Shen YH, Zhang C, Su J (2007). Triterpene saponins from *Bacopa monnieri* and their antidepressant effects in two mice models. J Nat Prod.

[43] Bilici M, Efe H, Köroglu MA, Uydu HA, Bekaroglu M, Deger O (2001). Antioxidative enzyme activities and lipid peroxidation in major depression: alterations by antidepressant treatments. J Affec Disord.

[44] Abdalla DS, Bechara EJ (1994). The effect of chlorpromazine and Li2CO3 on the superoxide dismutase and glutathione peroxidase activities of rat brain, liver and erythrocytes. Biochem Mol Biol Int.

[45] Khanzode SD, Dakhale GN, Khanzode SS, Saoji A, Palasodkar R (2003). Oxidative damage and major depression: the potential antioxidant action of selective serotonin re-uptake inhibitors. Redox Rep.

[46] Sairam K, Dorababu M, Goel RK, Bhattacharya SK (2002). Antidepressant activity of standardized extract of *Bacopa monniera* in experimental models of depression in rats. Phytomedicine.

[47] Bhattacharya SK, Bhattacharya A, Kumar A, Ghosal S (2000). Antioxidant activity of *Bacopa monniera* in rat frontal cortex, striatum and hippocampus. Phytother Res.

[48] Singh GK, Garabadu D, Muruganandam AV, Joshi VK, Krishnamurthy S (2009). Antidepressant activity of *Asparagus racemosus* in rodent models. Pharmacol Biochem Behav.

